# Persistent Luminescence in Eu^2+^-Doped Compounds: A Review

**DOI:** 10.3390/ma3042536

**Published:** 2010-04-06

**Authors:** Koen Van den Eeckhout, Philippe F. Smet, Dirk Poelman

**Affiliations:** Lumilab, Department of Solid State Sciences, Ghent University, Krijgslaan 281S1, 9000 Gent, Belgium; E-Mails: Philippe.Smet@UGent.be (P.F.S.); Dirk.Poelman@UGent.be (D.P.)

**Keywords:** persistent luminescence, europium, thermoluminescence

## Abstract

In 1996, Matsuzawa *et al*. reported on the extremely long-lasting afterglow of SrAl_2_O_4_:Eu^2+^ codoped with Dy^3+^ ions, which was more than 10-times brighter than the previously widely used ZnS:Cu,Co. Since then, research for stable and efficient persistent phosphors has continuously gained popularity. However, even today - almost 15 years after the discovery of SrAl_2_O_4_:Eu^2+^, Dy^3+^ - the number of persistent luminescent materials is still relatively low. Furthermore, the mechanism behind this phenomenon is still unclear. Although most authors agree on the general features, such as the existence of long-lived trap levels, many details are still shrouded in mystery. In this review, we present an overview of the important classes of known persistent luminescent materials based on Eu^2+^-emission and how they were prepared, and we take a closer look at the models and mechanisms that have been suggested to explain bright afterglow in various compounds.

## 1. Introduction

Persistent luminescence is an optical phenomenon, whereby a material is excited with high energy radiation (typically ultraviolet light, but other forms of energy such as beta rays can also be used) and the resulting visible luminescent emission remains visible for an appreciable time – from seconds to many hours - after the excitation has stopped. The effect is also called phosphorescence, afterglow, or LLP (short for Long Lasting Phosphorescence). As we will discuss further in this review, the long afterglow is governed by the slow liberation of trapped charge carriers by thermal excitation. Therefore, the process can be influenced by changing the temperature. Often, thermoluminescence - measuring the light output following the thermal release of trapped charges as a function of increasing temperature - is used as a diagnostic method for determining trap levels.

The phenomenon of persistent luminescence has been known to mankind for over a thousand years. Descriptions have been found of ancient Chinese paintings that remained visible during the night, by mixing the colors with a special kind of pearl shell [1]. The first scientifically described observation of persistent luminescence dates back to 1602, when shoemaker and alchemist Vincenzo Casciarolo discovered the famous Bologna stone. The curious glow of this stone was described by Fortunius Licetus in the *Litheosphorus Sive De Lapide Bononiensi* in 1640, and was most probably caused by barium sulfide present in the rock. Natural impurities in the stone were responsible for the long duration of the afterglow [1].

Until the end of the 20th century, very little research was done on the phenomenon of persistent luminescence. For many decades, zinc sulfide (ZnS) doped with copper (and later codoped with cobalt) was the most famous and widely used persistent phosphor [2,3]. It was used in many commercial products including watch dials, luminous paints and glow-in-the-dark toys. However, the brightness and lifetime that could be achieved with this material was rather low for practical purposes. To tackle this problem, traces of radioactive elements such as promethium or tritium were often introduced in the powders to stimulate the brightness and lifetime of the light emission [4]. But even then, a commercial glow-in-the-dark object had to contain a large amount of luminescent material to yield an acceptable afterglow.

**Figure 1:**
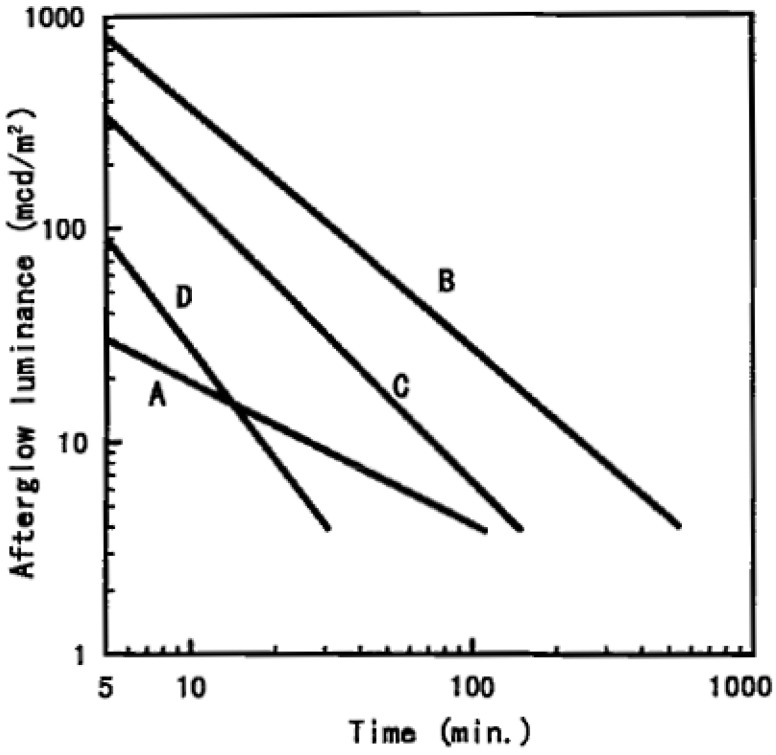
Comparison of afterglow characteristics measured after 10 min exposure to 200 lx of D_65_ light. A: SrAl_2_O_4_:Eu^2+^, B: SrAl_2_O_4_:Eu^2+^,Dy^3+^, C: SrAl_2_O_4_:Eu^2+^,Nd^3+^, D: ZnS:Cu,Co. (Reprinted with permission from [5]. Copyright 1996, The Electrochemical Society).

**Figure 2:**
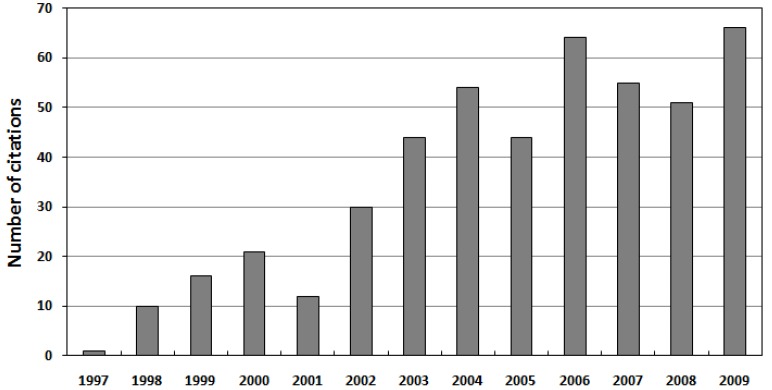
Number of citations of the 1996 paper by Matsuzawa *et al*. [5] according to the Web of Science.

In August 1996, Matsuzawa *et al*. published an article [5] that sent a shockwave through the until then relatively unpopular field of persistent luminescence. By codoping the green-emitting phosphor SrAl_2_O_4_:Eu^2+^ (already showing a relatively strong and long-lasting afterglow by itself [6]) with the rare earth element dysprosium (Dy^3+^), they were able to create a material that emitted bright light for hours after ending the excitation (simultaneously and independently, Takasaki *et al*. reported similar results [7]). They found an afterglow with both a far higher initial intensity and a much longer lifetime compared to ZnS:Cu,Co (Figure 1). Their discovery marked the beginning of a renewed search for different and better persistent luminescent materials. Initially, this research was concentrated on alkaline earth aluminates, and it took a few years before other types of compounds came into view. In 2001, Lin *et al*. reported a bright and long lasting afterglow in Sr_2_MgSi_2_O_7_:Eu^2+^,Dy^3+^ [8], and shortly afterwards other doped (di)silicates were found to exhibit an equally long afterglow. Today, almost 15 years after the discovery of SrAl_2_O_4_:Eu^2+^,Dy^3+^, the research for new persistent luminescent compounds has become increasingly popular (Figure 2). Quite surprisingly, the number of known compounds with a decent afterglow brightness and lifetime is still rather limited. In this review article, we will describe the major discoveries that have taken place during these last 15 years, and we will give an overview of the reported materials. We have limited ourselves to luminescence caused by Eu^2+^ ions, since these are the most common activators in persistent luminescent compounds. Section 2 of this review gives an overview of the materials studied.

Next, a short overview is presented of the most frequently used experimental techniques to estimate trap depths in persistent luminescent materials, by means of glow curve analysis or related methods (section 3).

In their famous article on SrAl_2_O_4_, Matsuzawa *et al*. suggested a mechanism to explain the occurrence of an efficient afterglow. This mechanism was initially accepted by most researchers, but a few others, such as Hölsä [9] and Dorenbos [10], raised questions about its validity. Other more complicated models were developed to tackle these revealed problems. Nowadays, most authors agree on the general idea behind persistent luminescence, but many details have yet to be clarified, and the discussion is ongoing. Therefore, we considered it useful to discuss and compare the different models that have been suggested by various authors. This is the subject of section 4 of the review.

## 2. Known Compounds

A wide variety of host materials are used as luminescent compounds, but when it comes to persistent luminescence, the number of known hosts is relatively low. The majority of research on this phenomenon is concentrated around the aluminates, with SrAl_2_O_4_ as most famous representative, and the silicates, represented by Sr_2_MgSi_2_O_7_. Besides these two main classes of materials, only few host crystals have been found to exhibit persistent luminescence with Eu^2+^ activators.

In this paragraph, we will give an overview of the compounds where Eu^2+^-based persistent luminescence has been reported. These materials are often labeled as ‘phosphorescent’, but the definition of phosphorescence is rather ambiguous, since the term is also used for luminescence where a quasi-stable state is involved, causing an increased lifetime of the fluorescence decay. However, even the decay from such a quasi-stable state usually does not last longer than a second [3]. We are interested in materials where the afterglow is caused by the existence of suitable charge carrier traps in the crystal [11], and remains visible for a reasonable amount of time. The borderline between ‘visible’ and ‘invisible’ is not sharply defined, and neither does there exist a consensus on a ‘reasonable amount of time’. In this review, we will focus on materials that have an afterglow decay time longer than several minutes, where the decay time is defined as the time between the end of the excitation and the moment when the light intensity drops below 0.32 mcd/m², roughly 100 times the sensitivity of the dark adapted human eye [12]. This is a definition similar to the one used in the safety signage industry, and by various researchers [13].

The data in the following tables are taken directly from the mentioned references. Only the codopants with the strongest positive influence on the afterglow are listed. Afterglow durations are meant to show the greater picture, and should only be seen as orders of magnitude.

New persistent materials are continuously discovered. The following list therefore does not pretend to be exhaustive, but it does, to the best of our knowledge, include nearly every host material in which a significant Eu^2+^-based afterglow has been reported.

### 2.1. Aluminates

Ever since the article by Matsuzawa *et al*. on SrAl_2_O_4_:Eu^2+^,Dy^3+^ [5], the aluminates have been the center of attention in persistent luminescent research, with a large number of publications (over 100 entries in the Web of Science up to December 2009). The aluminate compounds that are known to exhibit persistent luminescent properties are listed in Table 1.

The alkaline earth aluminates MAl_2_O_4_ (M = Ca,Sr,Ba) are by far the most studied family of persistent luminescent materials. The bright green luminescence of the monoclinic [14] SrAl_2_O_4_:Eu^2+^ was discovered in 1966 [15] and described by Blasse and Bril two years later, together with that of CaAl_2_O_4_:Eu^2+^ and BaAl_2_O_4_:Eu^2+^ [16]. It is interesting to note that, as mentioned before, even the non-codoped SrAl_2_O_4_:Eu^2+^ shows a considerable afterglow, indicating that the presence of codopants is not imperative to obtain persistent luminescence [6].

**Table 1:** Known Eu^2+^-based persistent luminescent aluminates.

Host material	Dopants	Fluorescence maximum (nm)	Afterglow maximum (nm)	Afterglow duration	References
SrAl_2_O_4_	Eu^2+^,Dy^3+^	520 (green)	idem	>30 h	[5,17]
CaAl_2_O_4_	Eu^2+^,Nd^3+^	440 (blue)	430 (blue)	>5 h	[18,19,20]
BaAl_2_O_4_	Eu^2+^,Dy^3+^	500 (green)	idem	>2 h	[21,22]
Sr_4_Al_14_O_25_	Eu^2+^,Dy^3+^	490 (blue)	idem	>20 h	[23,24,25]
SrAl_4_O_7_	Eu^2+^,Dy^3+^	480 (blue)	idem	>3 h	[26,27]
SrAl_12_O_19_	Eu^2+^,Dy^3+^	400 (blue)	idem	>3 h	[26]
Ca_12_Al_14_O_33_	Eu^2+^,Nd^3+^	440 (indigo)	idem	>10 min	[28]
Sr_3_Al_2_O_6_	Eu^2+^,Dy^3+^	510/610 (disputed)	idem	(disputed)	[29,30]
SrMgAl_10_O_17_	Eu^2+^,Dy^3+^	460 (blue)	515 (green)	>3 min	[31]
BaMgAl_10_O_17_	Eu^2+^,Co^3+^	450 (blue)	idem	>5 min	[32]

**Figure 3:**
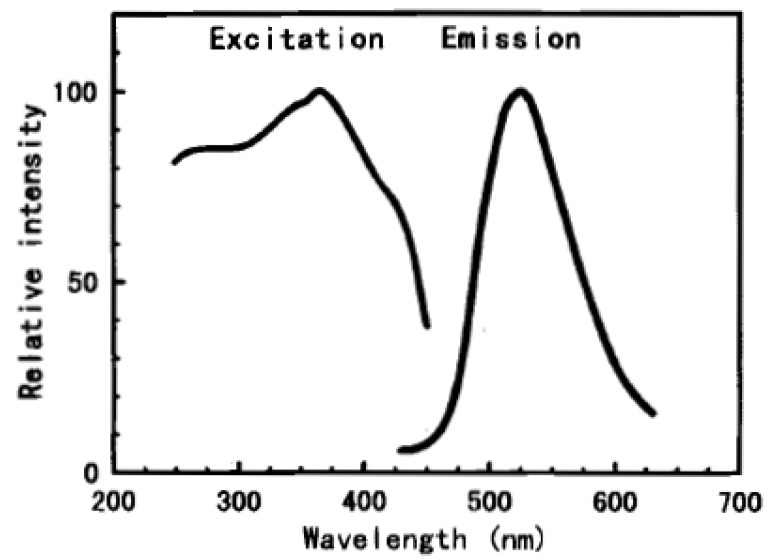
Excitation and emission spectra of SrAl_2_O_4_:Eu^2+^,Dy^3+^. (Reprinted with permission from [5]. Copyright 1996, The Electrochemical Society) .

Different paths were investigated to synthesize codoped MAl_2_O_4_:Eu^2+^ in an efficient, cheap and simple way. A solid-state reaction at 1300–1400 °C is most commonly used to obtain the desired compound, but also combustion [33,34,35,36,37], Pechini [33], microwave [38], laser heated pedestal growth (LHPG) [39] and sol-gel [40,41,42,43] methods were proven to be successful. However, it is worth noting that not all the techniques lead to identical crystallographic and luminescent properties. SrAl_2_O_4_:Eu^2+^,Dy^3+^ prepared by microwave synthesis shows a decreased initial brightness of the afterglow, together with a small blue shift of the emission spectrum, possibly due to the small grain size [38]. A similar blue shift is reported for sol-gel prepared SrAl_2_O_4_:Eu^2+^,Dy^3+^ [41,42,43]. During the preparation of CaAl_2_O_4_ by combustion or a sol-gel method, Hölsä and coworkers obtained an unusual hexagonal crystal structure instead of the expected monoclinic one [33,40]. Other researchers created grains with orthorombic structure [36]. It is clear that care should be taken when comparing luminescence of compounds prepared with different procedures.

The exact composition of the starting mixture has important consequences for the afterglow behavior. A deficit of alkaline earths usually enhances the afterglow [17], while an excess of barium in BaAl_2_O_4_:Eu^2+^,Dy^3+^ can annihilate the persistent luminescence completely [22].

**Figure 4:**
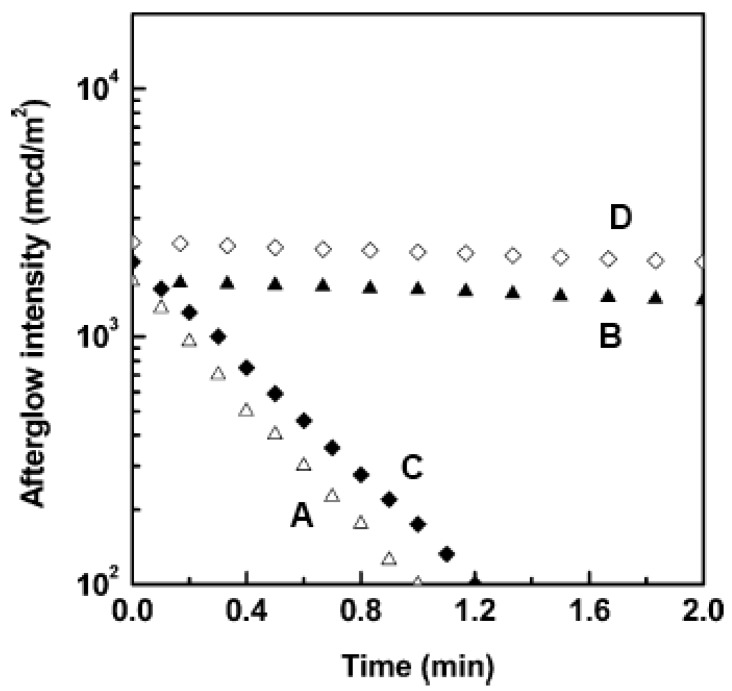
Afterglow decay curves for A: SrAl_2_O_4_:Eu^2+^,Dy^3+^, B: SrAl_2_O_4_:Eu^2+^,Dy^3+^ + B_2_O_3_, C: Sr_4_Al_14_O_25_:Eu^2+^,Dy^3+^ , D: Sr_4_Al_14_O_25_:Eu^2+^,Dy^3+^ + B_2_O_3_ (Reprinted with permission from [44]).

Several articles have been published on the influence of the ‘magic ingredient’ borate B_2_O_3_ on SrAl_2_O_4_:Eu^2+^,Dy^3+^. Usually, this material is added to the starting mixture as a flux agent [5], but it has other effects as well. Samples prepared without the addition of borate showed only very weak [41,44] or no persistent luminescence at all [45] (Figure 4), even though perfect SrAl_2_O_4_ phase formation was achieved. Apparently, the boron is incorporated in the host as BO_4_, where it forms substitutional defect complexes with Dy^3+^ [45]. This decreases the depth of the charge traps in SrAl_2_O_4_ from 0.79 eV to 0.65 eV, making it suitable for persistent luminescence at room temperature [44].

Another popular persistent luminescent aluminate is Sr_4_Al_14_O_25_:Eu^2+^,Dy^3+^, with a blue emission around 490 nm and an afterglow that remains visible for over 20 hours [23,24]. As in SrAl_2_O_4_, a small deficit of strontium enhances the persistent luminescence [46,47], and preparation without borate strongly reduced the afterglow [44] (Figure 4). Adding traces of silver ions (Ag^+^) increases the trap density and therefore has a positive influence on the afterglow [46].

The emission color of Sr_3_Al_2_O_6_: Eu^2+^,Dy^3+^ is indicated as ‘disputed’, because some confusing or contradictory results have been published. In triboluminescence measurements, the emission color is bright green [48] with a spectrum peaking around 510 nm, the same value was found in photoluminescence measurements by Chang *et al*. [29]. However, another research group reported red Eu^2+^-emission around 612 nm in the same material when prepared using microwave and sol-gel processes [30,49].

### 2.2. Silicates

The best known persistent luminescent silicate is Sr_2_MgSi_2_O_7_:Eu^2+^,Dy^3+^, first reported by Lin *et al*. in 2001 [8], but a long afterglow has also been discovered in a number of other silicate compounds (listed in Table 2).

**Table 2:** Known Eu^2+^-based persistent luminescent silicates.

Host material	Dopants	Fluorescence maximum (nm)	Afterglow maximum (nm)	Afterglow duration	References
Sr_2_MgSi_2_O_7_	Eu^2+^,Dy^3+^	470 (blue)	idem	>10 h	[8,50,51]
Ca_2_MgSi_2_O_7_	Eu^2+^(,Tb^3+^)	515/535 (green)	idem	>5 h	[52,53,54]
Ba_2_MgSi_2_O_7_	Eu^2+^,Tm^3+^	505 (green)	idem	>5 h	[55,56]
Sr_3_MgSi_2_O_8_	Eu^2+^,Dy^3+^	460 (blue)	idem	>10 h	[57,58]
Ca_3_MgSi_2_O_8_	Eu^2+^,Dy^3+^	470 (blue)	idem	>5 h	[57,59]
Ba_3_MgSi_2_O_8_	Eu^2+^,Dy^3+^	440 (blue)	idem	>1 h	[57]
CaMgSi_2_O_6_	Eu^2+^,Dy^3+^	445 (blue)	idem	>4 h	[54,60]
Sr_3_Al_10_SiO_20_	Eu^2+^,Ho^3+^	465 (blue)	idem	>6 h	[61,62]
CaAl_2_Si_2_O_8_	Eu^2+^,Dy^3+^ or Pr^3+^	435 (blue)	435/510 (blue)	>3 h	[63,64]
Sr_2_Al_2_SiO_7_	Eu^2+^,Dy^3+^	485 (blue/green)	idem	>2 h	[65]
Sr_2_ZnSi_2_O_7_	Eu^2+^,Dy^3+^	460 (blue)	idem	>5 min	[66,67]
Sr_2_SiO_4_	Eu^2+^,Dy^3+^	480 (green)	idem	>5 min	[68]

The family of materials M_2_MgSi_2_O_7_ (M = Ca,Sr,Ba), also called alkaline earth akermanites, plays a role similar to that of MAl_2_O_4_ in the aluminate group. They are often used as an example material when presenting afterglow mechanisms, and they are the most widely studied persistent luminescent silicates.

A solid-state reaction at 1200–1400 °C is the most common way to prepare M_2_MgSi_2_O_7_ samples, but recently sol-gel [69], co-precipitation [70] and combustion methods [71,72] were also applied successfully.

Curiously, Hölsä and coworkers found that the afterglow in Ca_2_MgSi_2_O_7_:Eu^2+^ was significantly reduced upon addition of trivalent rare earth ions (except for Tb^3+^, with a weak positive influence, Figure 4) [52]. This is in sharp contrast with the huge enhancement of the afterglow in nearly all other codoped aluminates and silicates. For example, Sr_2_MgSi_2_O_7_:Eu^2+^,Dy^3+^ [73] and Ba_2_MgSi_2_O_7_:Eu^2+^,Tm^3+^ [55] have a much brighter and longer afterglow than their non-codoped variants. 

**Figure 5:**
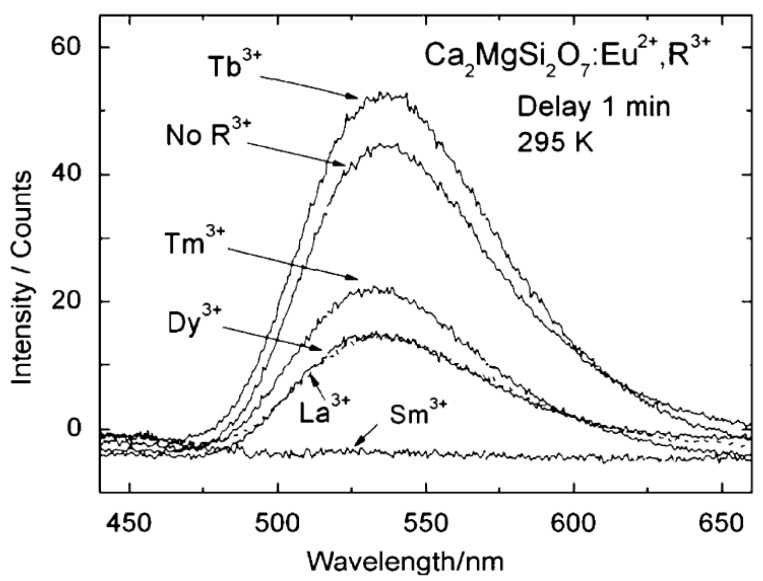
Persistent luminescence spectra of selected Ca_2_MgSi_2_O_7_:Eu^2+^,R^3+^ materials at 295 K. (Reprinted with permission from [52]).

Another remarkable observation of Ca_2_MgSi_2_O_7_:Eu^2+^ was a disagreement on the emission spectrum. Blasse [53] and Hölsä [52] found an emission maximum around 535 nm (Figure 5), whereas Mao and coworkers reported an emission band centered around 515 nm when codoping with Dy and Nd [54,74,75].

Before we end this paragraph on silicates, it is worth mentioning a publication by Wang *et al*. on Sr_2_ZnSi_2_O_7_:Eu^2+^,Dy^3+^ prepared with a sol-gel method [67]. By applying different synthesis temperatures, they were able to obtain grains of various sizes, and they showed that a smaller grain size enhanced both the brightness and lifetime of the afterglow.

### 2.3. Other compounds

In addition to the discussed aluminates and silicates, only few compounds are known to exhibit persistent luminescence (Table 3). Many of these originate from LED research and are also commonly used as conversion phosphors.

**Table 3:** Other known Eu^2+^-based persistent luminescent compounds.

Host material	Dopants	Fluorescence maximum (nm)	Afterglow maximum (nm)	Afterglow duration	References
CaS	Eu^2+^,Tm^3+^(,Ce^3+^)	650 (red)	idem	>1 h	[78,79,80]
CaGa_2_S_4_	Eu^2+^,Ho^3+^ or Ce^3+^	555 (yellow)	idem	>30 min	[81,82,83]
Ca_2_SiS_4_	Eu^2+^,Nd^3+^	660 (red)	idem	>30 min	[84]
Sr_2_P_2_O_7_	Eu^2+^,Y^3+^	420 (blue)	idem	>8 h	[85]
Ca_2_P_2_O_7_	Eu^2+^,Y^3+^	415 (blue)	idem	>6 h	[86]
SrMg_2_P_2_O_8_	Eu^2+^,Ce^3+^	400 (blue)	idem	>2 h	[87]
Ca_2_Si_5_N_8_	Eu^2+^,Tm^3+^	610 (orange)	620 (orange)	>1 h	[88,89]
CaAl_2_B_2_O_7_	Eu^2+^,Nd^3+^	465 (blue)	idem	>1 h	[90]
SrB_2_O_4_	Eu^2+^	430 (blue)	idem	unknown	[91]

The first class of materials appearing in this table is the sulfides. These are hygroscopic, and therefore less stable than the aluminates and silicates, but they generally show an increased red shift compared to their oxide counterparts, enabling emission at longer wavelength, in the yellow, orange or red region of the visible spectrum. This increased red shift is mainly determined by a larger centroid shift for sulfides than oxides, which is due to a larger covalency between the anion and the Eu^2+^ ion [76].

CaS is a well-known phosphor host, and upon doping with Bi^3+^ blue persistent luminescence is obtained [77]. However, Jia *et al*. achieved a red afterglow when doping with Eu^2+^ and Tm^3+^ [78,79]. Additionally, they proved that traces of Ce^3+^ further enhanced the lifetime and brightness of the afterglow [80].

Phosphates are another class of materials with the possibility of exhibiting afterglow. Pang *et al*. recently reported persistent luminescence in two similar orthorhombic pyrophosphate compounds, α-Ca_2_P_2_O_7_:Eu^2+^ [86] and α-Sr_2_P_2_O_7_:Eu^2+^ [85], codoped with Y^3+^ ions. Ca_2_P_2_O_7_:Eu^2+^,Mn^2+^ had already drawn some attention as a possible conversion phosphor in white LEDs, due to the presence of both a blue Eu^2+^-based and an orange Mn^2+^-based emission band [92]. Pang *et al*. found that by codoping with Y^3+^-ions, the blue europium emission remained visible for over six h in Ca_2_P_2_O_7_:Eu^2+^,Y^3+^ and over even eight h in Sr_2_P_2_O_7_:Eu^2+^,Y^3+^.

The family of alkaline earth nitrido-silicates M_2_Si_5_N_8_:Eu^2+^ (M = Ca,Sr,Ba) is also widely used in white LED research. They not only have a very broad excitation spectrum extending into the visible part of the spectrum, but also a very efficient yellow (M = Ba), orange (M = Ca) or orange-red (M = Sr) emission [93]. Furthermore, they are very stable against moisture and heat. In 2009, it was shown by Van den Eeckhout *et al*. that Ca_2_Si_5_N_8_:Eu^2+^ exhibits a weak intrinsic persistent luminescence that can be greatly enhanced by codoping with different rare earth ions. Codoping with Tm^3+^ ions yields the best results with a persistent lifetime of over one h [89]. Simultaneously, Miyamoto *et al*. independently reported the same results. Furthermore, they replaced part of the Ca atoms by Sr to obtain a more reddish color. They concluded that around 10% of the Ca should be substituted by Sr to have an optimal trade-off between emission intensity and color [88].

### 2.4. Dopant and codopant concentrations

A question that arises when investigating persistent luminescent compounds is the doping rates of Eu^2+^ and the rare earth codopants that should be applied in order to achieve the longest and most intense afterglow possible. In general, these values differ from the ones in fluorescence. For example, in SrAl_2_O_4_, Wang *et al*. found optimal fluorescence intensity with 6.6% of Eu^2+^ doping (*i.e.,* 6.6% of the Sr sites in the host crystal is occupied by a Eu^2+^ ion) [4], while Matsuzawa *et al*. suggested 1% of Eu^2+^ and 2% of Dy^3+^ to obtain the brightest afterglow [5]. Similarly, Zhao *et al*. reported the brightest persistent luminescence in CaAl_2_O_4_ upon doping with 0.5% of Eu^2+^ and 1% of Nd^3+^, while the fluorescence of this sample was worse than that of a similar sample with 1% of Eu^2+^ and 1% of Nd^3+^ [37].The matter is further complicated by the fact that the optimal doping rates vary for different compounds and different codopants. Upon codoping of SrAl_2_O_4_:Eu^2+^ with Dy^3+^, around 1% of Eu^2+^ and 2% of the codopant is preferred, as previously mentioned. The optimal concentration for Nd^3+^ as a codopant, on the other hand, is around 1%, *i.e.,* the same concentration as the Eu^2+^ ions [5].

Most authors choose to follow these typical concentrations of 1% Eu^2+^ and 1 or 2% RE^3+^, but only rarely is it verified (or at least reported) that these are indeed the optimal values. Lin *et al*. confirmed the ideal 2/1 ratio of Dy/Eu ions in Sr_4_Al_14_O_25_ [23], and in Ca_2_MgSi_2_O_7_:Eu^2+^,Dy^3+^,Nd^3+^ Jiang *et al*. found an optimal afterglow at a Dy/Eu ratio of around 20/7 [75]. Some materials show somewhat atypical behavior, for example, Sabbagh Alvani *et al*. report an optimal Dy/Eu ratio of 1/2 in Sr_3_MgSiO_8_:Eu^2+^,Dy^3+^ [58].

## 3. Estimating Trap Depths

Charge carrier traps play a crucial role in all the suggested persistent luminescence mechanisms. One of their main properties is their ‘depth’, the activation energy needed to release a captured charge carrier. Shallow traps (with a depth lower than around 0.4 eV [94]) are fully emptied at low temperatures, and do not actively take part in processes at room temperature. Very deep traps (around 2 eV or deeper [10]), on the other hand, require more energy to be emptied than is available at room temperature. Therefore, charge carriers caught by these traps remain there until the material is sufficiently heated. To observe persistent luminescence at room temperature, the traps should have an appropriate activation energy somewhere between these two extremes (a trap depth around 0.65 eV is considered to be optimal [5]). 

In chapter 4, we will see that the nature of the trapped charge carriers (electrons or holes) is still subject of discussion. It is therefore noteworthy that the techniques described in the following paragraphs give an estimate for the trap depth regardless of the charge carrier type.

### 3.1. Thermoluminescence

The experimental technique of thermoluminescence (TL) was first explored in the beginning of the 20th century [95]. In this method, a material is initially heated or kept in the dark for a sufficiently long time until all traps are emptied. The material is subsequently cooled to liquid nitrogen or helium temperatures, and fully excited by a (usually white) light source for some time. The excitation is switched off, and the temperature is increased linearly, with a heating rate *β* (in K/s). Meanwhile, the optical emission from the sample is measured and plotted against temperature. The curve obtained in this way is usually denounced as ‘glow curve’ [3,11]. It is customary to also measure the temperature dependence of the fluorescence (by repeating the measurement under constant excitation), in order to compensate for temperature quenching effects [3].

**Figure 6:**
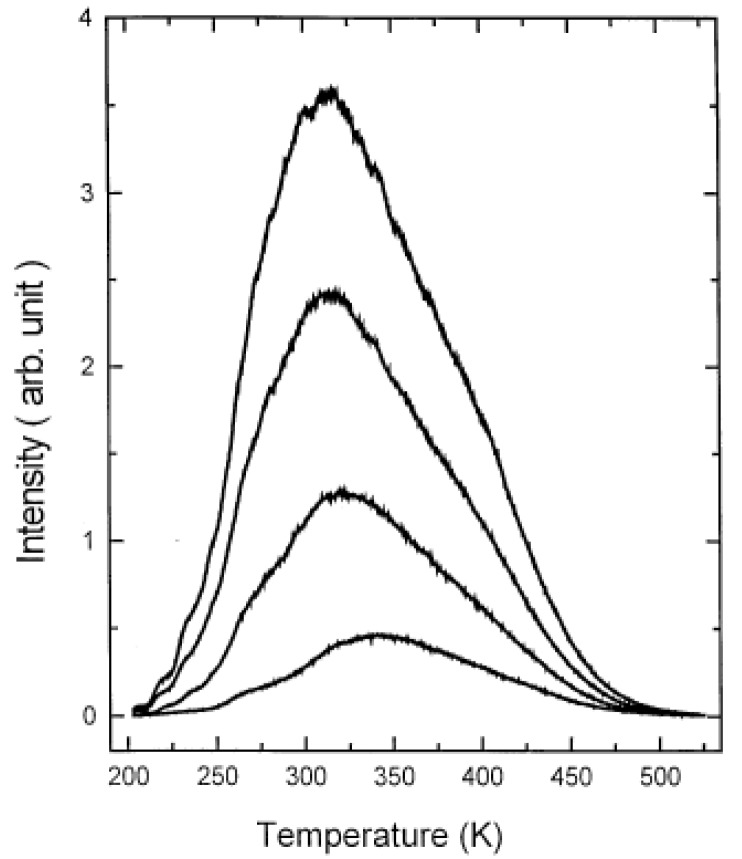
The glow curve for SrAl_2_O_4_:Eu^2+^,Dy^3+^ after excitation for different periods of time. From bottom to top: 10, 30, 60 and 120 seconds (Reprinted with permission from [39]).

For nearly all materials, the glow curve shows one or more broad, often asymmetrical peaks (an example for SrAl_2_O_4_:Eu^2+^,Dy^3+^ is given in Figure 6). Each peak is believed to originate from a separate trap or trap distribution. Studying the shape and location of these different peaks can provide insight in the different depths and distributions of the traps present in the sample. These studies are especially popular in the field of geology, where thermoluminescence is used as a dating technique [95], and radiation dosimetry [96]. Thermoluminescence measurements and glow curves are often neglected in the study of persistent luminescence, but also in this field they can be useful to give information on trap depths and distributions.

Many authors have tried to develop a method to analyze glow curves in a reliable and consistent way. A full discussion of all these efforts and their theoretical details is beyond the scope of this text, but can be found in, for example, [95]. We will briefly mention those methods that are still used frequently today. The most simple way to estimate trap depths from the location of the glow peak maximum was derived empirically and formulated in 1930 by Urbach [97]. If *T_m_* is the temperature for which the glow curve reaches a maximum, the related trap depth is approximately:

(1) $$E_{T}=T_{m}/500$$

This equation, despite its simplicity, incorporates an important intuitive result: deeper traps (*i.e.,* with a higher activation energy *E_T_*) result in glow curve peaks at higher temperature. Indeed, to free charge carriers from deeper traps, a larger thermal energy is required. The trap energy obtained is only approximate, since equation (1) is not based on a theoretical model for the behavior of charge carriers in materials with trap levels. This problem was tackled in a famous series of articles by Randall and Wilkins in 1941 [98,99]. They looked at the simplified situation of a host material with a single trap level in the band gap. It is important to note that although they assumed the charge carriers to be electrons, their results are equally valid in the case of holes. According to their theory, the glow intensity *I* during heating is found to be proportional to the concentration *n*, the frequency factor or ‘escape frequency’ *s* of the trapped charge carriers and an exponential part containing the trap depth (Boltzmann factor):

(2) $$I\propto sn\cdot \exp\left(-\frac{E_{T}}{kT}\right)$$

where *k* is Boltzmann*’*s constant. This leads to a transcendental equation for the trap depth:

(3) $$\frac{\beta E_{T}}{kT_{m}^{2}}=s\cdot \exp\left(-\frac{E_{T}}{kT_{m}}\right)$$

where *T_m_* is again the location of the glow curve maximum, and *β* is the heating rate (in K/s). This linear relationship between glow intensity and trapped charge carrier concentration is generally referred to as “first order kinetics”. This theory assumes that every charge carrier released from a trap recombines in a luminescent center. The possibility of `retrapping*’*, when the charge carrier is caught again by a trap and not by a luminescent center, is assumed to be negligible. However, Randall and Wilkins pointed out that certain experimental results suggested similar probabilities for both processes (retrapping and recombination) [99]. In 1948, Garlick and Gibson (coworkers of Randall and Wilkins) explored this possibility and obtained a “second order kinetics”, with the glow intensity proportional to *n²*. They found that this assumption yielded better results for several materials [100]. The effect of the parameters *s*, *T*, *E_T_* and *β* on the shape of the glow curve can be found in [101], illustrated for both first and second order kinetics. 

Two major obstacles arise when applying the Randall-Wilkins or associated methods. Firstly, the frequency factor *s* is initially unknown. Very often, *s* is approximated or assumed comparable to the vibrational frequency of the lattice. The value of *s* does, however, greatly influence the resulting trap depth. Even worse, the frequency factor itself can (and most probably does) depend on temperature, and therefore changes during the course of the thermoluminescence experiment, as pointed out by for example Chen [102]. The uncertainty on the value of *s* is bypassed in the Hoogenstraaten method [103]. For this, the thermoluminescence experiment is repeated several times for different heating rates *β_i_*. According to equation (3), the exact glow maximum will shift to different temperatures *T_mi_* when the heating rate is varied. For every value of *β_i_*, a similar equation can be written down, and the unknown *s* can thus be eliminated by plotting ln($T_{mi}^{2}$/*β_i_*) *versus* 1/*T_mi_* and fitting these data points with a straight line. The slope of this line reveals the activation energy of the trap [3].

A second problem is the unknown ‘order’ of the glow curve under investigation. Some glow peaks yield better fitting results when first order kinetics are assumed, some require second order kinetics for a decent fit. This problem can be avoided by looking only at the low-temperature side of the observed glow peaks. Regardless of the order of the peak, the intensity will be proportional to an exponential Boltzmann factor [3]:

(4) $$I\propto \exp\left(-\frac{E_{T}}{kT}\right)$$

The estimation of trap depths by fitting the low-temperature end of a glow peak to such an exponential factor is known as the “initial rise” method. In practice, however, it is often very difficult to isolate the initial rising part of a glow peak, making the obtained trap depths less accurate.

Another popular way to avoid the problem of the unknown order was proposed by Chen in 1969 [102] and is known as ‘general order kinetics’. Chen looked more closely at the shape of the glow peak, by taking into account the full width ω at half maximum and its low-temperature and high-temperature halves τ and δ, respectively. He was able to write down several compact formulas to calculate the trap depth in specific cases. Furthermore, he calculated the coefficients necessary for these formulas for many different values of the trap depth (0.1–1.6 eV) and frequency factor (10^5–^10^13^ s^-1^) [104]. For example, the activation energy for a peak with first order kinetics and a temperature-independent frequency factor is simply given by:

(5) $$E_{T}=2kT_{m}\left(\frac{1.26T_{m}}{\omega }-1\right)$$

All of the mentioned models are still in use today. To simplify glow curve analysis for researchers, specialized software was developed, such as *TL Glow Curve Analyzer* [105], which makes use of first, second and general order kinetic equations [106].

The analysis of glow curves has fascinated researchers for many decades, but it should be approached with caution. The results obtained by the different techniques described above are not always comparable. This is illustrated by applying several methods on the same glow curve, as was done by for example [107] in the case of cerium and copper doped barium sulfide. In this paper, the Urbach, Randall-Wilkins and Chen methods (among others) were compared. The Randall-Wilkins analysis gives the lowest trap depth (around 0.45 eV), Chen*’*s method results in a trap approximately 0.05–0.10 eV higher, and Urbach*’*s formula estimates a trap depth of about 0.75 eV. This indicates that comparing different trap depths should only be done when both glow curves were studied with the same technique. Another problem that often occurs in practice is the overlap between different peaks, which can make decent analysis almost impossible.

### 3.2. Other methods

Thermoluminescence is the most common way to estimate trap depths, but some other techniques are known that do not rely on the analysis of glow curves. Bube [108] noted that, for first order kinetics, the temperature dependence of the afterglow decay constant is given by:

(6) $$\tau \propto s^{-1}\exp\left(\frac{E_{T}}{kT}\right)$$

By measuring the decay constant for various temperatures and plotting the results in an Arrhenius diagram, a straight line is obtained whose slope is related to the trap depth. Often, the afterglow decay cannot be described by a single decay constant. In that case, multiple exponentials can be fitted to the decay, and for each of these an appropriate trap depth can be estimated. Although this method is sometimes used in binary sulfides [107,109], it is, to the best of our knowledge, never applied in Eu^2+^-doped materials.

Another technique was proposed by Nakazawa and is frequently called “transient thermoluminescence” (TTL,TRL) [110]. While the sample is heated very slowly, it is repeatedly excited by a light source. The intensity of the afterglow is measured at various delay times *t_d_* after the termination of the excitation. When this intensity is plotted against the temperature, the location of the peak *T_m_* depends on the delay time in the following way:

(7) $$t_{d}\propto s^{-1}\exp\left(\frac{E_{T}}{kT_{m}}\right)$$

An advantage of the TTL method is that it is unaffected by thermal quenching, as opposed to normal thermoluminescence [110].

Table 4 shows some approximate values for the best-known persistent luminescent materials, as reported in literature. It should be noted that these are not always exact results. Sometimes the trap is not a single level, but a distribution of energy levels. In that case, a mean value of the trap is cited. All trap levels mentioned in the respective articles are noted, although sometimes they are too shallow or too deep to contribute to the persistent luminescence at room temperature. The numbers in the table demonstrate again that comparing trap depth values estimated with different techniques should be done with caution.

**Table 4:** Estimated trap depth(s) in some of the best-known persistent luminescent materials.

Phosphor	Method	Trap depths (eV)	Reference
SrAl_2_O_4_:Eu^2+^,Dy^3+^	initial rise	0.55/0.60/0.65/0.75	[111]
	Chen	0.30/0.65/0.95/1.20/1.40	[112]
	Hoogenstraaten	0.65	[5]
	TTL	1.1	[113]
CaAl_2_O_4_:Eu^2+^,Nd^3+^	initial rise	0.55/0.65	[114]
Sr_4_Al_14_O_25_:Eu^2+^,Dy^3+^	Chen	0.72	[47]
	Chen	0.49	[46]
	TTL	0.91	[25]
Sr_2_MgSi_2_O_7_:Eu^2+^,Dy^3+^	Chen	0.75	[51]
Sr_2_MgSi_2_O_7_:Eu^2+^,Nd^3+^	Hoogenstraaten	0.08/0.18/0.29/0.23	[115]
Ca_2_MgSi_2_O_7_:Eu^2+^,Tm^3+^	Chen	0.56	[52]

## 4. Suggested Persistent Luminescence Mechanisms

The discovery of the persistent luminescent properties of SrAl_2_O_4_:Eu^2+^,Dy^3+^ also marked the beginning of a renewed search for the underlying mechanisms. Until then, relatively little research had been done on this subject. It was generally agreed that after excitation, charge carriers could get caught by so called ‘traps’, energy levels inside the forbidden band gap with a very long lifetime. The charge carriers are only gradually released from these traps, after which they can return to the activators and produce luminescence. Quite some research had been done on thermoluminescence glow curves and how to extract information about trap depth from them. However, details such as the nature and origin of the traps and the charge carriers were still unclear.

However, since 1996, different mechanisms have been suggested, ranging from very basic conceptual models to complex systems with multiple charge traps of various types and depths. In the following paragraphs, we will try to give a brief but adequate overview of the most important ones, how they were conceived and how they were justified or disproved by experimental results.

### 4.1. The Matsuzawa model

**Figure 7:**
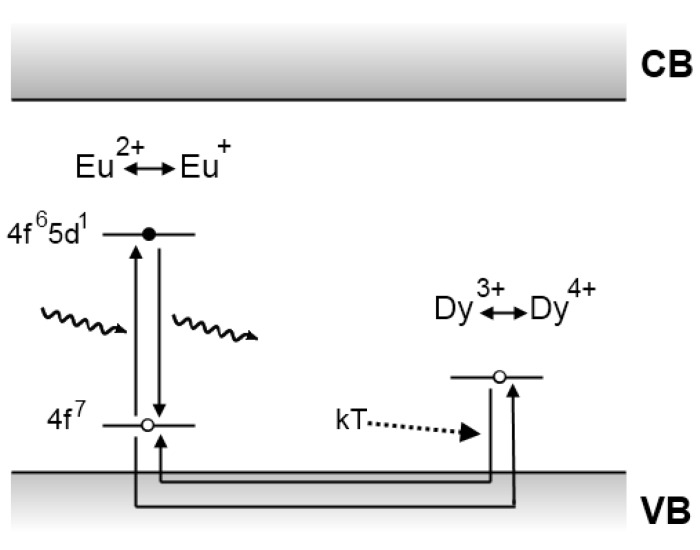
Persistent luminescence mechanism proposed by Matsuzawa *et al*. for SrAl_2_O_4_:Eu^2+^,Dy^3+^ [5].

In the same famous article announcing the discovery of SrAl_2_O_4_:Eu^2+^,Dy^3+^, Matsuzawa *et al*. tried to explain the origins of the extraordinary persistent luminescence. A schematic picture of their model is shown in Figure 7.

In this model, holes are assumed to be the main charge carriers. This assumption is based on earlier measurements by Abbruscato on non co-doped SrAl_2_O_4_:Eu^2+^, which also shows a weak afterglow. From his results obtained by Hall measurements, Abbruscato concluded that holes in the valence band had to be the main charge carriers [6]. He suspected that Sr^2+^ vacancies acted as traps for these holes. Additionally, Matsuzawa *et al*. performed non-uniform illumination photoconductivity measurements, which also suggested that holes are the main charge carriers [5].

The Matsuzawa model modified Abbruscato*’*s assumptions in order to explain the influence of rare earth codoping. When an Eu^2+^ ion is excited by an incident photon, there is a possibility that a hole escapes to the valence band, thereby leaving behind a Eu^+^ ion. The hole is then captured by a trivalent rare earth ion, such as Dy^3+^, thus creating a Dy^4+^ ion. After a while, thermal energy causes the trapped hole to be released into the valence band again. From there it can move back to a Eu^+^ ion, allowing it to return to the Eu^2+^ ground state with emission of a photon [5].

The Matsuzawa model quickly gained popularity [22,45,116,117,118], and was used frequently to explain observed afterglow in newly discovered compounds [23,54,59]. Various thermoluminescence [39,113,119,120], photoconductivity [118] and electron paramagnetic resonance [121] measurements were performed to confirm the validity of the model. However, the results of these experiments were often inconclusive and no hard evidence for the model could be found. It was inevitable that certain researchers started to raise questions about the Matsuzawa mechanism.

### 4.2. The Aitasalo model

**Figure 8:**
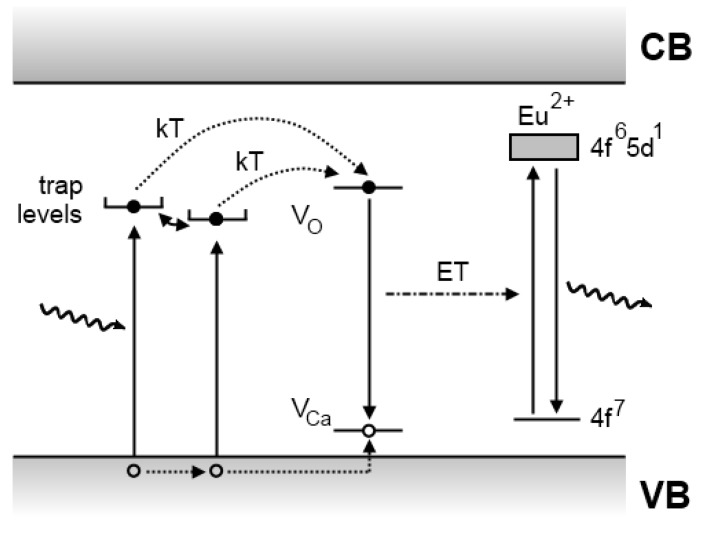
Persistent luminescence mechanism proposed by Aitasalo *et al*. for CaAl_2_O_4_:Eu^2+^,Dy^3+^ [122].

In 2003, Aitasalo *et al*. suggested a model that differed considerably from the Matsuzawa model (Figure 8) [122]. In this model, electrons are excited directly from the valence band into trap levels of unspecified origin. The hole that is created in this way migrates towards a calcium vacancy ($V_{Ca}^{\prime \prime }$ in the Kröger-Vink notation [123]) where it is caught. The electron is removed from the trap level by thermal energy and ends up at an oxygen vacancy level. Since the conduction band is located too high above the energy level of the oxygen vacancy trap to enable a thermally assisted transition to the conduction band, they assumed that the energy released on recombination of the electron and the hole was delivered directly to the europium ions, by means of energy transfer. This assumption requires close proximity of the vacancies to the luminescent centers. The transferred energy excites an electron of europium to a 5d level, followed by recombination and emission of the persistent luminescent light [122]. It should be noted that only holes are present as free charge carriers (in the valence band), which explains the previous observations by Abbruscato and Matsuzawa.

Hölsä and coworkers introduced this model for several reasons. Firstly, the Matsuzawa model ignored the observed persistent luminescence in non-codoped SrAl_2_O_4_:Eu^2+^ [124]. Therefore, a model that avoided the explicit use of the trivalent rare earth codopants needed to be developed. Aitasalo *et al*. explained the influence of the codopants by suggesting that they increased the number of lattice defects, because the trivalent lanthanide ions occupy the divalent alkaline earth sites, leading to spontaneous defect creation for charge compensation. This also explains why adding Sm^3+^ to the material is detrimental for the persistent luminescence, since the Sm^3+^ is reduced to Sm^2+^ during preparation, thereby removing the cation vacancies, which act as hole traps [122].

A second reason for rejecting the original Matsuzawa model was the implausibility of the occurrence of monovalent europium and tetravalent dysprosium ions in the material. Aitasalo *et al*. argued that the reduction of Eu^2+^ to Eu^+^ and the oxidation of Dy^3+^ to Dy^4+^ would result in chemically unstable ions [122]. This reasoning was later supported by other authors such as Dorenbos [10]. 

A final observation that encouraged Aitasalo *et al*. to suggest a new persistent luminescence mechanism was the observation that the 430 nm persistent luminescence of CaAl_2_O_4_:Eu^2+^,Nd^3+^ could be induced by excitation with wavelengths as large as 530 nm [9]. They concluded that the absorption of two photons had to occur in the process, through direct excitation of an electron from the valence band into a long-lived intermediate trap level, followed by an excited state absorption (ESA). Since the Matsuzawa model assumed that the trapped charge carriers originated from the Eu^2+^ ions, it could not explain how these could be created with such low-energy photons.

### 4.3. The Dorenbos model

Dorenbos put great effort into the determination of lanthanide energy levels in inorganic compounds, with applications in scintillator physics and persistent luminescence [125]. As previously mentioned, he agreed with Aitasalo *et al*. that the existence of Eu^+^ and Dy^4+^ in aluminate or silicate compounds is highly improbable [10]. Secondly, he pointed out that the assumed hole on the ground state of Eu^2+^ after excitation is based on faulty reasoning. The energy levels of the lanthanides are localized, in contrast to the delocalized Bloch states of the valence and conduction band. Therefore, the 4f state of europium after the excitation should not be interpreted as a ‘real hole’ that can accept an electron. He was not convinced by the observation of hole conduction by Abbruscato and Matsuzawa, and noted that more detailed research was required [10]. 

**Figure 9:**
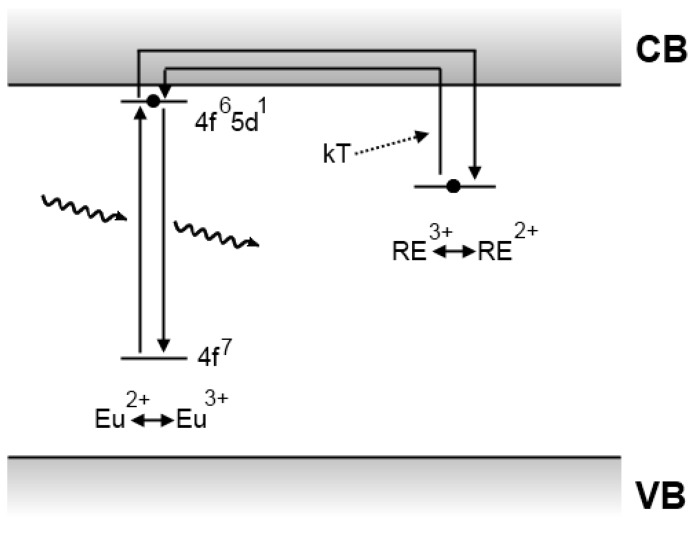
Persistent luminescence mechanism proposed by Dorenbos *et al*. for aluminate and silicate compounds [10].

These problems with the Matsuzawa model encouraged Dorenbos to present a different model in 2005, depicted in Figure 9. As in Matsuzawa’s model, electrons are excited in divalent europium ions. Since the 5d level of divalent europium lies very close to the conduction band [10], these excited electrons can easily be released into the conduction band and subsequently caught by a trivalent rare earth codopant, creating a divalent ion. Thermal energy can then release the trapped electron, after which it recombines upon reaching a luminescent center [10,126]. This mechanism is basically the same as the one suggested by Matsuzawa, but it does not require the existence of Eu^+^ and RE^4+^. It can, however, not explain the existence of intrinsic persistent luminescence in non-codoped materials.

Based on his previous research on the location of lanthanide levels in inorganic compounds, Dorenbos derived that the energy level of Dy^2+^ (*i.e.,* Dy^3+^ after capture of an electron) in SrAl_2_O_4_ lies approximately 0.9 eV below the conduction band [10], which is of the same order as the trap depth found in SrAl_2_O_4_:Eu^2+^,Dy^3+^ [5,111,113]. The Dorenbos model also explains why Sm^3+^ and Yb^3+^ strongly reduce the afterglow. Previous work revealed that the relevant levels of Sm^2+^ and Yb^2+^ are located much lower than those of the other divalent rare earth ions such as Dy^2+^ and Nd^2+^ [127]. This results in traps that are too deep to be emptied at room temperature.

### 4.4. The Clabau model

Around the same time as Dorenbos, Clabau *et al*. reviewed the existing mechanisms for persistent luminescence and found that a revision was needed. For the same reasons as Dorenbos, these authors did not accept the Matsuzawa model. Furthermore, they mention EPR measurements that show a decrease in the Eu^2+^ concentration during excitation, followed by an increase as soon as the excitation is terminated, continuing until the afterglow ends. They concluded that Eu^2+^ must participate in the trapping process, which contradicted the idea of energy transfer to Eu^2+^ after the trapping, as suggested by Aitasalo [13,128,129].

**Figure 10:**
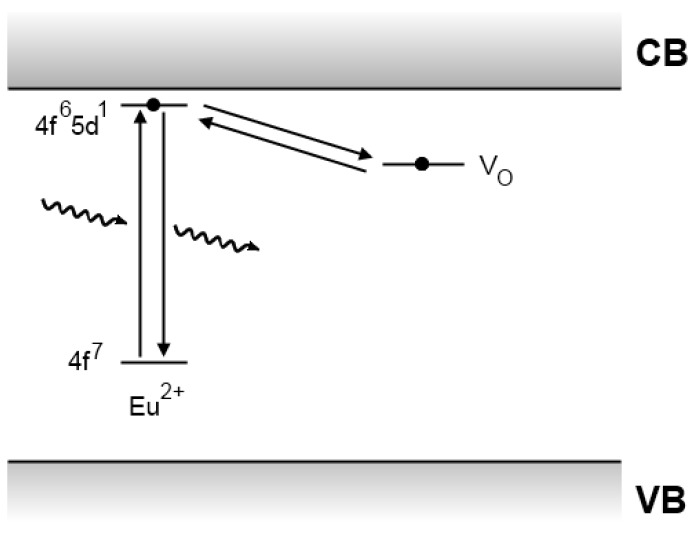
Persistent luminescence mechanism proposed by Clabau *et al*. for SrAl_2_O_4_:Eu^2+^,Dy^3+^ [128].

The model proposed by Clabau *et al*. is shown in Figure 10. It is similar to the Dorenbos model, but differs on some important points. Firstly, there is no migration of electrons through the conduction band. The transport of electrons between the traps and the luminescent centers is believed to occur through direct transfer, which requires close proximity between the europium ions and the lattice defects [128]. This assumption is based on measurements of the photoconductivity in SrAl_2_O_4_:Eu^2+^,Dy^3+^ under UV excitation, which increases up to 250 K, and subsequently enters a plateau phase until 300 K, indicating that no free charge carriers are released around this temperature. However, thermoluminescence measurements around 300 K clearly show the presence of de-trapping processes at this temperature (Figure 6). From this, Clabau *et al*. concluded that the interaction between the traps and the luminescent centers could not occur via the conduction band.

A second difference to the Dorenbos mechanism is the nature of the traps. By comparing glow curves of non-codoped and Dy^3+^-codoped SrAl_2_O_4_:Eu^2+^, Clabau *et al*. noticed that the relevant peaks differed in size and location, but were very similar in shape. From this, they concluded that the chemical nature of the trap was not influenced under codoping. This led them to the idea that lattice defects, namely oxygen vacancies, must act as traps in SrAl_2_O_4_:Eu^2+^,RE^3+^ [13].

The influence of the lanthanides as codopants is explained by their stabilizing influence on the oxygen vacancies. The ionization potentials of the rare earths can be used as a measure for the extent of this stabilization, since a lower ionization potential will cause the codopant to attract oxygen vacancies more strongly, hereby increasing the trap depth [129]. Indeed, when codoping SrAl_2_O_4_:Eu^2+^ with different rare earths with an increasing ionization potential, the duration of the afterglow is shortened [128].

### 4.5. Recent developments

**Figure 11:**
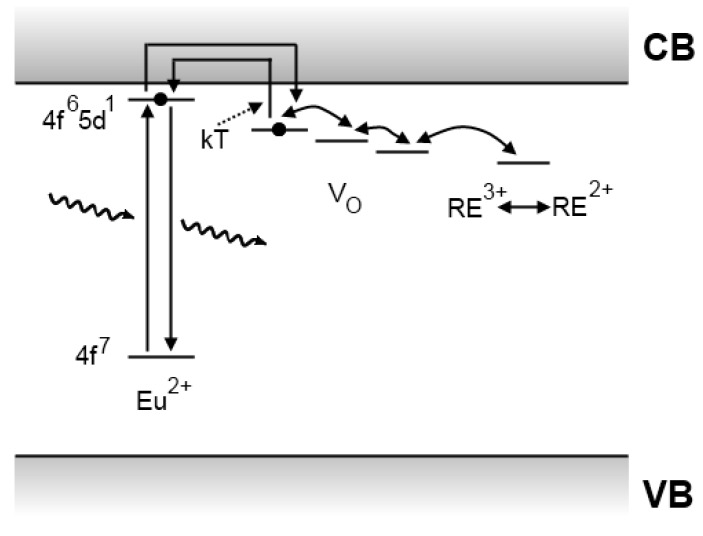
Persistent luminescence mechanism proposed in 2006 by Aitasalo *et al*. for CaAl_2_O_4_:Eu^2+^,Dy^3+^ [114].

In 2006, Aitasalo *et al*. described a mechanism for persistent luminescence that incorporates suggestions from both Clabau and Dorenbos (Figure 11) [114]. Electrons that are excited in the Eu^2+^ luminescent centers can easily escape into the conduction band. Both oxygen vacancies and trivalent codopant ions introduce trap levels, but the exact nature was not clarified, since these defects can interact with each other and form complex aggregates [114]. When enough thermal energy is available, the captured electrons can escape again into the conduction band and recombine in a luminescent center.

### 4.6. Experimental evidence

Synchrotron radiation measurements offer interesting new ways to study persistent luminescence, and were not always fully appreciated until now. Qiu *et al*. [130], Qi *et al*. [131], and recently Carlson *et al*. [132] performed X-ray absorption near edge structure (XANES) measurements to uncover the valence of the rare earth ions in Sr_2_MgSi_2_O_7_:Eu^2+^,RE^3+^. Both divalent and trivalent europium were encountered, but for the rare earth codopants only the trivalent form could be detected. The divalent form predicted by Dorenbos could not be identified. No monovalent europium or tetravalent codopant ions, as would be expected in the Matsuzawa model, were observed. This could indicate that the Matsuzawa and Dorenbos models are not suitable. However, it could also be due to a low concentration of filled trap levels in these materials, which makes it hard to detect these specific valence states. Indeed, none of the models described above gives information about the actual number or concentration of trap levels and trapped charge carriers involved in the afterglow.

Electron paramagnetic resonance (EPR) measurements are another suitable way to study traps in the investigated materials. Hölsä *et al*. used EPR to prove the existence of electrons in anion vacancies (*i.e.,* F^+^ colour centers) in non-codoped and even non-Eu^2+^-doped CaAl_2_O_4_ [133].

**Figure 12:**
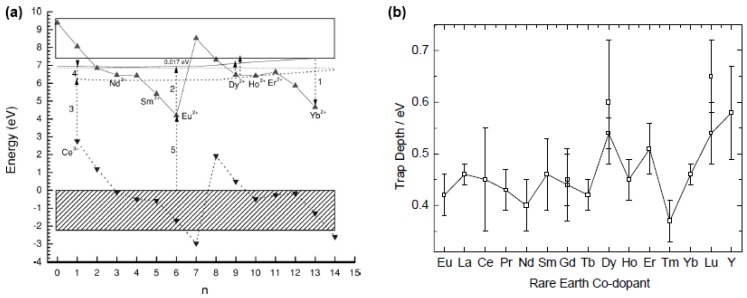
(a) Typical energy level pattern for the lanthanide series (n = number of 4f electrons) in SrAl_2_O_4_:Eu^2+^,RE^3+^(Reprinted with permission from [10]. Copyright 2005, The Electrochemical Society) (b) Trap depths estimated with the Hoogenstraaten method for different codopants in CaAl_2_O_4_:Eu^2+^,RE^3+^ (Reprinted with permission from [122])

Dorenbos showed that the 4f levels of the lanthanide series follow a characteristic pattern relative to each other, independent of the host material (Figure 12a) [127]. If the trivalent codopants indeed act as traps, as the Dorenbos model claims, it is reasonable to expect that this pattern can be recognized by studying the trap depth for different codopants. Unfortunately, results on this matter are scarce and rather ambiguous. Aitasalo *et al*. estimated the trap depth for the entire lanthanide series as codopant, but did not find a clear trend (Figure 12b) [122]. The results obtained by Van den Eeckhout *et al*. in Ca_2_Si_5_N_8_:Eu^2+^,RE^3+^ seem to confirm the Dorenbos trend, but were not yet performed for the entire rare earth series [89].

**Figure 13:**
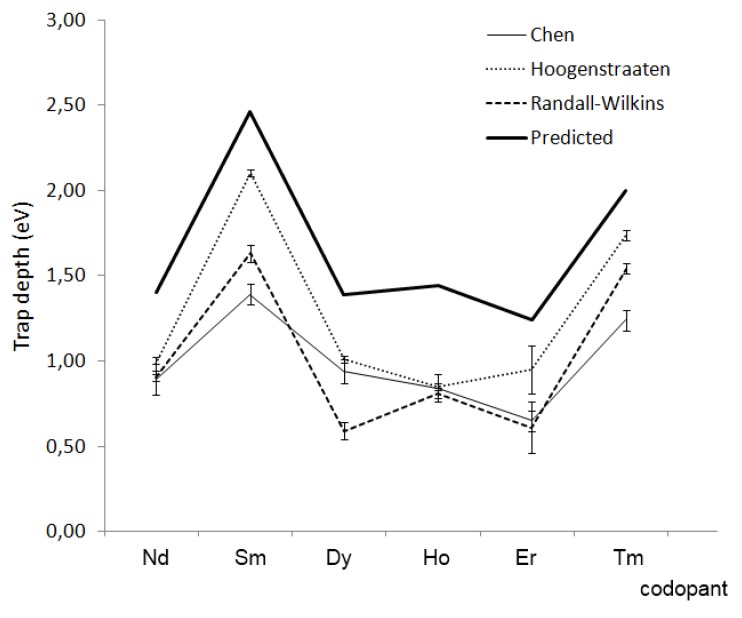
Trap depths in YPO_4_:Ce^3+^ codoped with various lanthanides, as predicted by the Dorenbos energy level scheme and estimated from thermoluminescence measurements (data taken from [134]).

For the case of YPO_4_:Ce^3+^,RE^3+^, a material used in thermoluminescence dosimetry, Bos *et al*. measured glow curves for different lanthanide codopants [134]. The trap depths obtained in this way (estimated using the different analysis techniques discussed earlier) are shown in Figure 13, together with the predicted depth using the energy level scheme by Dorenbos. Although this is not a Eu^2+^-based compound, these results seem to confirm that the codopant ions play the role of traps in at least some materials.

We can conclude that experimental backup for the different suggested models is very scarce and often indecisive. Further measurements are vital to unravel the mysteries surrounding the persistent luminescence mechanism.

### 4.7. Concluding remarks

The exact mechanisms governing persistent luminescence in materials have yet to be clarified. Intense research by several groups has produced different models, but none of these have enough experimental backup to be identified as the true afterglow mechanism. Further research, both theoretical and experimental, remains vital. The Matsuzawa model has by now lost a lot of its popularity, because of some flaws pointed out by several authors. The influence of lattice defects such as oxygen vacancies cannot be neglected, given the afterglow in non-codoped compounds, but it remains unclear if a similar reasoning can be used to explain persistent luminescence in other host materials such as the sulfides or nitrides.

It is nowadays generally assumed that the main charge carriers are electrons. This is similar to earlier models developed for binary sulfides such as ZnS:Cu [103]. Other sulfides were also interpreted with electron trapping, for example in CaGa_2_S_4_:Eu^2+^,Ce^3+^ [83] and CaS:Eu^2+^,Tm^3+^ [78] (although for the latter a hole trapping mechanism has also been suggested [79]). However, it remains unclear how one should interpret the results found by Abbruscato and Matsuzawa that point in the direction of holes.

One question that still needs to be answered concerns the excitation spectrum of the persistent luminescence. This is not necessarily the same as the excitation spectrum of the fluorescence (see, for example, [84]), however, it is often overlooked in research on persistent luminescent materials. The Aitasalo model, where electrons are excited directly from the valence band into a trap level, leaves room for differences between fluorescence and persistent luminescence excitation spectra. However, in the other models, both should be similar, since the charge carriers are always created in the luminescent europium ions, both for fluorescence and persistent luminescence. For this reason, additional research on persistent luminescence excitation spectra could deliver further insight into the underlying mechanisms [84]. Care should be taken, however, as recording excitation spectra for persistent phosphors is not straightforward [135].

## 5. Challenges and Perspectives

The discovery of SrAl_2_O_4_:Eu^2+^,Dy^3+^ and Sr_2_MgSi_2_O_7_:Eu^2+^,Dy^3+^ has placed the Eu^2+^-doped materials in the epicenter of persistent luminescent research. Their brightness and very long lifetime completely overshadows that of their most important predecessor, ZnS:Cu,Co. The quest for new persistent luminescent materials has resulted in several blue and green emitting persistent phosphors that remain visible for many hours or even up to an entire day.

Can we expect the discovery of new and even better persistent luminescent materials in the (near) future? On the one hand, this is doubtful. The past 15 years of intense research have brought us only a handful of phosphors that are bright enough to consider their use in practical applications. Furthermore, these materials almost exclusively fall into two main categories, the aluminates and the silicates, which have already been explored intensely. On the other hand, recent developments on some other material groups, such as the sulfides, phosphates and nitrides, have shown that persistent luminescence is not limited to specific hosts. It is noteworthy that the majority of these ‘special’ new phosphors are originally conversion phosphors for LEDs. The number of known host materials for LED phosphors is large [136,137] and many of these could be promising persistent phosphors. More specifically, codoping some of these LED phosphors with rare earth ions could deliver propitious results.

When looking at the known Eu^2+^-doped persistent luminescent compounds, earlier in this text, the lack of yellow, orange and red phosphors is striking. This dearth has two major causes. Firstly, it is difficult to obtain a high enough crystal field in oxides for Eu^2+^ to emit radiation in the red region of the visible spectrum [76]. To deal with this, we can look at other luminescent centers (such as, for example, Eu^3+^ in the famous red persistent phosphor Y_2_O_2_S:Eu^3+^,Mg^2+^,Ti^4+^ [138,139] or Mn^2+^ in BaMg_2_Si_2_O_7_ [140]) or we can turn to other host materials (such as the aforementioned sulfides or nitrides).

A second major problem with red persistent luminescent materials is the phenomenon known as the Purkinje effect [141]. It is well known that, in daylight, the human eye is most sensitive for green light, and less for red and blue wavelengths. In the dark, this eye sensitivity shifts to shorter wavelengths, thus making it easier to see blue light, but almost impossible to detect dim orange or red light. This shift in the eye sensitivity is maximal for luminous intensities lower than 1 mcd/m² (the scotopic region), but the reduced perception of red light already starts at intensities lower than 1 cd/m² (commonly called the mesopic region [142,143]). As persistent luminescent materials usually operate in this mesopic region, this means that a red phosphor has to be more intense, compared to a blue or green one, to achieve the same perceived brightness [12]. This makes the search for bright red persistent luminescent compounds particularly hard.

At present, the mechanisms responsible for persistent luminescence are not yet fully understood. Most authors agree on the general mechanism of charge carriers getting trapped in long-lived energy levels inside the band gap. Many details, however, remain unclear. For example, it is unknown whether these charge carriers originate from the luminescent centers, or if they are created directly by excitation from the valence or conduction band. Another obscurity is how the energy stored in these traps is conducted to the luminescent centers, by direct energy transfer, or through charge carrier transport in the conduction or valence band. The influence of codopants and lattice defects in the neighborhood of the activators is another unsolved issue. Future experiments are necessary to unravel these mysteries. The most promising techniques are the ones that were, until recently, often overlooked. Synchrotron radiation (such as XANES and EXAFS) and EPR measurements offer insight into the structure, composition, valence states and charge distribution of materials, and could provide an answer to these theoretical questions, answers that cannot be offered by photoluminescence and thermoluminescence experiments only.

Persistent luminescent research has a promising future. The search for new and better materials with Eu^2+^ ions as activators continues and has recently turned to other host materials, based on the developments in LED conversion phosphors. Additionally, the quest to unravel the mechanism behind the persistent luminescence has entered a new path. Various models have been proposed in the past few decades with only a small amount of experimental backup, but only recently researchers have started applying new and promising techniques that could confirm or disprove these theories. A better understanding of the exact mechanism is crucial for the development of practical applications such as emergency signs [144], traffic signage, dials and displays, textile printing, medical diagnostics [145], and more. Eu^2+^ activated long-lasting phosphors will play a vital role in the bright future of persistent luminescence.
